# Teen beauty trends: The ethics and impact of aesthetic medicine on adolescents

**DOI:** 10.1016/j.jpra.2025.10.014

**Published:** 2025-10-16

**Authors:** Marco Borin, Giulio Nittari, Rebecca Susanna Degliuomini, Luigi Schiraldi, Pietro Giovanni Di Summa, Giovanni Buzzaccarini

**Affiliations:** a UO.C. Otorhinolaryngology, ASST Grande Ospedale Metropolitano Niguarda Piazza Ospedale Maggiore, 20162 Milan, Italy; b Telemedicine and Telepharmacy Centre, University of Camerino, Via Madonna Delle Carceri 9, 62032 Camerino, Italy; c Artemisia Clinic, via Giacomo Leopardi 1, 20123 Milan, Italy; d Plastic and Hand Surgery Department, Lausanne University Hospital (CHUV), Rue du bugnon 46, Lausanne 1011, Switzerland; e Department of Obstetrics and Gynaecology, IRCCS San Raffaele Scientific Institute, Vita-Salute San Raffaele University, Via Olgettina 58-60, Milan 20132, Italy

**Keywords:** Adolescents, Aesthetics, Ethics, Social Media, Self-esteem

Dear Editor,

The increasing prevalence of aesthetic medicine among adolescents poses critical ethical, medical, and psychological questions. Social media-driven beauty standards compel young individuals to seek treatments for their beautification.^1^ The recent ISAP 2024 Survey highlighted the distribution of aesthetic procedures performed on individuals aged 17 or younger, revealing a notable prevalence of interventions at a stage of ongoing physical and psychological development. Rhinoplasty (i.e. non-surgical nose reshaping) emerges as the most common procedure, with 45,512 cases representing 4.2 % of the total, followed by botulinum toxin injections (39,440 cases, 0.5 %) and liposuction (22,959 cases, 1.1 %). Additionally, breast augmentation (13,269 cases, 0.8 %), non-surgical fat reduction (13,354 cases, 1.9 %), and external genital surgery (7097 cases, 1.9 %) are also performed within this age group.^2^ While these procedures may enhance confidence, they involve substantial risks, particularly for those subjects still maturing emotionally and physically.^3^ On this regard, our letter to editor aims to raise awareness and provoke a call for regulation in aesthetic medicine treatments in adolescents.

First, many teenagers seek cosmetic interventions to address insecurities or conform to peer pressure. However, these procedures may only serve as temporary fixes for deeper emotional issues such as low self-esteem or body dysmorphia, well documented in literature. Moreover, the pervasive use of filters on social media, often promoting unrealistic and idealized beauty standards, is reshaping adolescents' self-perception, contributing to body dissatisfaction, and driving a concerning rise in the demand for aesthetic procedures among younger populations. This raises concerns about the adolescents' ability to make fully informed decisions about their appearance.^4^ From a psychological perspective, there is caution that cosmetic treatments could strengthen the harmful belief that appearance dictates self-worth. Adolescence is a formative period for self-esteem, and reliance on aesthetic procedures may hinder adolescents from accepting their natural appearance.

For these reasons and secondly, parental involvement is crucial (both in positive or negative way). While some parents support their child's choice, others may unknowingly reinforce societal pressures.

Thirdly, the triadic figure involved is the healthcare provider (HCP). Medical professionals advise that purely cosmetic procedures for minors should be approached with caution unless a clear benefit is evident. A thorough evaluation, often including mental health consultations, is essential to ensure that the adolescent’s decision is well-considered and not influenced by external pressures.^3^^,^^4^ Supporters argue that, when conducted responsibly, aesthetic treatments can benefit adolescents by improving confidence, particularly for those with physical features that affect their self-esteem.^5^ Minimally invasive procedures are considered lower risk and can alleviate emotional distress. However, these interventions should be approached by doctors conservatively, with a full understanding of the potential long-term consequences.

Considering the above three actors, ethical approach advocates for a thorough consultation process to determine if the procedure is genuinely in the adolescent's best interest. While parental guidance and clinical oversight remain essential in decisions concerning adolescent cosmetic procedures, these individual-level factors are insufficient when considered in isolation. Personal choices are invariably influences by broader structural contexts (i.e., social norms, regulatory landscapes, and the pervasive influence of social media). Taken this into consideration, it becomes imperative to advocate for systemic interventions that extend beyond the clinical encounter: school-based programs that foster healthy body image; the implementation of age-appropriate regulatory guidelines for aesthetic medicine treatments; and targeted social media rule initiatives to mitigate external pressures from predator aesthetic medicine influencer HCP.^5^ Addressing these structural dimensions not only enhances protective frameworks but also shifts the conversation from reactive regulation to proactive education and prevention.

## Declaration of competing interest

Pietro Giovanni Di Summa is a Deputy Editor for JPRAS.
